# Dexamethasone ameliorates severe pneumonia but slightly enhances viral replication in the lungs of SARS-CoV-2-infected Syrian hamsters

**DOI:** 10.1038/s41423-021-00793-7

**Published:** 2022-01-05

**Authors:** Lunzhi Yuan, Ming Zhou, Jian Ma, Xuan Liu, Peiwen Chen, Huachen Zhu, Qiyi Tang, Tong Cheng, Yi Guan, Ningshao Xia

**Affiliations:** 1 State Key Laboratory of Molecular Vaccinology and Molecular Diagnostics, National Institute of Diagnostics and Vaccine Development in Infectious Diseases, School of Life Sciences, School of Public Health, Xiamen University, Xiamen, Fujian China; 2 State Key Laboratory of Emerging Infectious Diseases, School of Public Health, Li Ka Shing Faculty of Medicine, The University of Hong Kong, Hong Kong, SAR China; 3 Guangdong-Hong Kong Joint Laboratory of Emerging Infectious Diseases/Joint Laboratory for International Collaboration in Virology and Emerging Infectious Diseases, Joint Institute of Virology (STU/HKU), Shantou University, Shantou, Guangdong China; 4 EKIH Pathogen Research Institute, Futian District, Shenzhen, Guangdong China; 5 Department of Microbiology, Howard University College of Medicine, Washington, DC USA; 6 Research Unit of Frontier Technology of Structural Vaccinology, Chinese Academy of Medical Sciences, Xiamen, Fujian China

**Keywords:** Infection, Infectious diseases, Immunotherapy

The pandemic caused by severe acute respiratory syndrome coronavirus 2 (SARS-CoV-2) has resulted in more than 230 million cases and over four million deaths worldwide. Furthermore, multiple emerging SARS-CoV-2 variants have shown enhanced infectivity, transmissibility, pathogenicity and ability to escape neutralization by vaccine-induced humoral immunity [1]. The antibody resistance of SARS-CoV-2 variants constitutes a challenge for current vaccines and therapeutic antibodies. No specific antiviral is currently available for coronavirus in humans [2]. Although remdesivir was approved by the FDA for the treatment of SARS-CoV-2 infection, the therapeutic effect is limited, particularly for critical cases with severe pneumonia. Therefore, a more effective anti-SARS-CoV-2 regimen is needed to end the COVID-19 pandemic.

SARS-CoV-2-induced immunological disorder is the leading cause of severe pneumonia and death in critical cases. After SARS-CoV-2 infection, excessive and imbalanced immune responses result in dysregulated secretion of proinflammatory cytokines, such as tumor-necrosis factor α (TNF-α), interferon γ (IFN-γ), interleukin 6 (IL-6) and IL-10, which largely increase the severity of pneumonia and lead to multiorgan failure. The hyperactivated and dysregulated immune system in critical cases necessitates anti-inflammatory immunotherapy [3]. Recently, several clinical studies demonstrated that the FDA-approved glucocorticoid drug dexamethasone is able to reduce disease severity and mortality in hospitalized human patients with SARS-CoV-2 infection [4, 5]. In contrast to newly developed drugs, dexamethasone has unique advantages, including being inexpensive and widely available and having 60 years of safety profiling [6]. However, the mechanism of the effects of dexamethasone treatment on SARS-CoV-2-induced severe pneumonia is not clear. Moreover, the side effects, intervention time point, and duration of dexamethasone treatment need further evaluation in clinical studies and animal models.

To mimic patients with severe pneumonia caused by SARS-CoV-2, Syrian hamsters were intranasally infected with 1 × 10^4^ plaque-forming units (PFUs) of an ancestral SARS-CoV-2 strain (AP-8) as previously described [7, 8]. SARS-CoV-2-infected hamsters were untreated or treated with 1, 3, or 5 doses of dexamethasone (1 mg/kg per dose) via intraperitoneal injection (Fig. 1a). SARS-CoV-2-infected hamsters without dexamethasone treatment (control group) exhibited progressive mean body-weight loss of up to 13.4 ± 1.8% from 1 to 7 days post infection (dpi) (Fig. 1b). However, SARS-CoV-2-infected hamsters treated with 1, 3, or 5 doses of dexamethasone exhibited body-weight loss of 9.8 ± 2.1%, 7.1 ± 1.5% and 1.9 ± 2.3% at 7 dpi, respectively (Fig. 1b). To evaluate the lung-pathogenesis severity, viral load, and host immune response, all of the hamsters were euthanized at 7 dpi. Lung lobes were collected and fixed in formalin for systematic pathological analysis. Hematoxylin and eosin (H&E) staining of lung lobes revealed that in contrast to the noninfected (mock) hamsters, SARS-CoV-2-infected hamsters without dexamethasone treatment had typical features of severe pneumonia, including increased lung-lobe consolidation and alveolar destruction, diffusive inflammation, protein-rich fluid exudate, hyaline-membrane formation and severe pulmonary hemorrhage (Fig. 1c and Supplementary Fig. 1). H&E staining of the lung lobes of SARS-CoV-2-infected hamsters treated with dexamethasone showed alleviation of the lung pathological changes (Fig. 1c and Supplementary Fig. 1). Notably, diffusive lung injury was not observed at 7 dpi in the lung lobes of SARS-CoV-2-infected hamsters treated with five doses of dexamethasone (Fig. 1c and Supplementary Fig. 1). In addition, the severity of lung pathogenesis was quantified by a comprehensive pathological score based on alveolar septum thickening and consolidation, hemorrhage, exudation, pulmonary edema and mucus, inflammatory-cell recruitment and infiltration among all of the hamster lung lobes (Fig. 1d and Table S1).

**Fig. 1:**
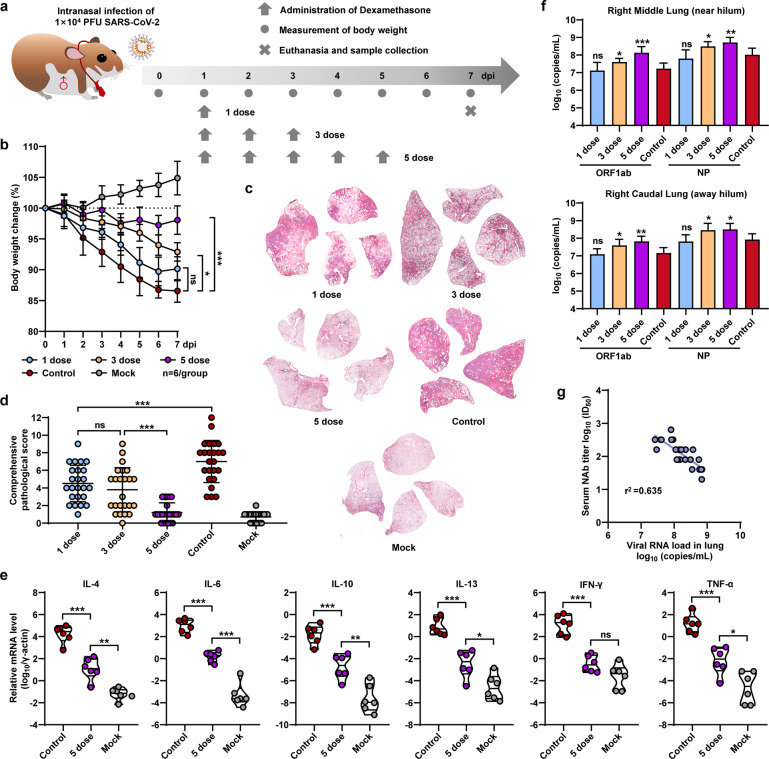
Detection of physiological and lung pathological changes in SARS-CoV-2-infected hamsters treated with dexamethasone. **a** Schematic diagram of SARS-CoV-2 infection and animal operation. Male hamsters were intranasally inoculated with 1 × 10^4^ PFU of SARS-CoV-2 and then received intraperitoneal injections of 1, 3, and 5 doses of dexamethasone. Body weight was observed daily. Animals were euthanized at 7 dpi for virological and histological analysis. SARS-CoV-2-infected hamsters were used as the control group. The hamsters without SARS-CoV-2 infection were set as the mock group. **b** Body-weight changes with SARS-CoV-2 infection from 0 to 7 dpi (*n* = 6). **c** Representative H&E staining of lung-lobe sections collected from SARS-CoV-2-infected and mock hamsters at 7 dpi. H&E staining for all the remaining hamsters were shown in Supplementary Fig. S1. **d** Comprehensive pathological scores for lung sections were determined based on the severity and percentage of injured areas for each lung lobe. **e** Fold changes in the mRNA levels of proinflammatory cytokines in lung tissues collected from SARS-CoV-2-infected hamsters treated with five doses of dexamethasone at 7 dpi (*n* = 6). The mRNA levels of proinflammatory cytokines were standardized to the housekeeping gene γ-actin. **f** Viral RNA levels in the right middle-lung tissue (near hilum) and right caudal-lung tissue (away from the hilum) collected from hamsters at 7 dpi were measured by RT–PCR (*n* = 6). Primers for the SARS-CoV-2 ORF1ab and NP genes were used. **g** The linear relationship between serum antibody levels and average lung viral RNA levels of each individual hamster.

Current evidence suggests that dysregulated production of proinflammatory cytokines plays an important role in the immunopathology progression of SARS-CoV-2 infection [9]. To determine whether dexamethasone is able to suppress excessive proinflammatory cytokine expression after SARS-CoV-2 infection, the mRNA levels of several proinflammatory cytokines in homogenized lung tissues collected at 7 dpi were measured by real-time polymerase chain reaction (RT–PCR). Compared with the mock hamsters, the SARS-CoV-2-infected hamsters showed an approximately 1000- to 50,000-fold increase in the mRNA levels of IL-4, IL-6, IL-10, IL-13, TNF-α and IFN-γ in homogenized lung tissues collected at 7 dpi (Fig. 1e). However, these increased mRNA levels of proinflammatory cytokines, which are related to cytokine storms, were significantly suppressed by dexamethasone treatment (Fig. 1e). Therefore, inhibition of excessive proinflammatory cytokine expression may be a promising strategy to relieve the severe pneumonia caused by SARS-CoV-2 infection.

Next, we analyzed viral replication in respiratory-tract organs, including the turbinate, trachea and lung by RT–PCR, which amplified SARS-CoV-2 open-reading frame 1ab (ORF1ab) and nucleocapsid protein (NP) for detection of viral RNA load in the homogenized tissues collected at 7 dpi. Compared with SARS-CoV-2-infected hamsters without dexamethasone treatment, 5-dose dexamethasone-treated hamsters had higher levels of viral RNA in the turbinate, trachea, and middle and caudal lung (Fig. 1f and Supplementary Fig. 2), but only the increases in the lungs were statistically significant. To test whether dexamethasone treatment is able to alter antibody responses, we analyzed the levels of specific antibodies against the SARS-CoV-2 receptor-binding domain (RBD) and the neutralizing-antibody (NAb) titers in the serum of hamsters at 7 dpi. In brief, SARS-CoV-2-infected hamsters treated with dexamethasone showed lower serum anti-RBD antibody and NAb titers (Supplementary Fig. 3) than those not treated. The decrease in antibody levels in serum was related to the duration of dexamethasone treatment, suggesting a high correlation between the increase in viral RNA in lung tissue and treatment with dexamethasone. Indeed, the average viral RNA load in lung tissues had an inverted proportional relationship with the NAb titer at 7 dpi (Fig. 1g). These results demonstrated that dexamethasone slightly enhances viral replication in lung tissues because of attenuation of antibody responses. In addition, the D614G mutation in the SARS-CoV-2 variant enhanced its pathogenicity in hamsters. Fortunately, five doses of dexamethasone were adequate to rescue the infected hamsters from death (Supplementary Fig. 4 and Table S2).

The progression of SARS-CoV-2-induced severe pneumonia is driven by virus replication-induced immunopathology. Therefore, both antiviral and anti-inflammatory countermeasures are important for the clinical care in critical cases. The overarching findings of this study are that the immunosuppressive properties of dexamethasone may be a double-edged sword for therapy for SARS-CoV-2 infection: suppression of inflammation but enhancement of viral replication. In SARS-CoV-2-infected hamsters, dexamethasone treatment induced attenuation of serum-neutralizing antibody and RBD-specific antibody titers, which resulted in a slight enhancement of viral replication in the lung. To offset this drawback, it is necessary to combine dexamethasone treatment with antiviral agents such as remdesivir and potent antibody cocktails. In the hamster model, dexamethasone has been demonstrated to be an efficient anti-inflammatory agent to treat SARS-CoV-2-induced severe pneumonia (Supplementary Fig. 5). In addition, our data highlighted that the timing and duration of dexamethasone treatment might have an impact on its therapeutic effect. Overall, we revealed the advantages and disadvantages of dexamethasone treatment, which may guide its clinical application in the foreseeable future.

## Supplementary information

Supplementary materials
