# Supplementary material for: Dexamethasone ameliorates severe pneumonia but slightly enhances viral replication in the lungs of SARS-CoV-2-infected Syrian hamsters
Source: Cell Mol Immunol. 2022 Jan 5;19(2):290–2. doi: 10.1038/s41423-021-00793-7 (PMC8727236; doi:10.1038/s41423-021-00793-7)
Supplement: Supplementary file 1 — Supplementary materials [file 41423_2021_793_MOESM1_ESM.docx]

**Supplementary materials**

**Dexamethasone ameliorates severe pneumonia but slightly enhances viral replication in lung of SARS-CoV-2-infected Syrian hamster**

**Methods**

**Experimental Animal and Biosafety**

The Golden Syrian Hamster was raised in the specific pathogen free animal feeding facilities. All the animal experiments were approved by the Medical Ethics Committee (SUCM2021-112). All experiments with infectious SARS-CoV-2 were performed in the biosafety level 3 (BSL-3) and animal biosafety level 3 (ABSL-3) facilities affiliated to the State Key Laboratory of Emerging Infectious Diseases, School of Public Health, The University of Hong Kong. Our staff wear powered air-purifying respirators that filter the air, and disposable coveralls when they culture the virus and handle animals that are in isolators. The researchers are disinfected before they leave the room and then shower on exiting the facility. All facilities, procedures, training records, safety drills, and inventory records are subject to periodic inspections and ongoing oversight by the institutional biosafety officers who consult frequently with the facility managers.

**Preparation of Virus Stock**

The SARS-CoV-2 ancestral strain AP-8 (hCoV-19/China/AP8/2020; GISAID accession number: EPI_ISL_1655937) and the D614G mutation virus strain AP-62 (hCoV19/China/AP62/2020; GISAID accession number: EPI_ISL_2779639) were passaged on Vero cells. Viral stocks were prepared in Vero cells (#CCL-81, ATCC) with DMEM containing 2% FBS, 5ug/mL TPCK-trypsin, Penicillin-Streptomycin and 30mmol/L MgCl_2_ (#11995, #10270106, #T1426 and #15140-122; purchased from GIBCO, SIGMA-ALDRICH and Invitrogen). Viruses were harvested and stored in ultra-low temperature refrigerator. The titers were determined by means of plaque assay in Vero cells.

**Virus Inoculation and Sample Collection**

The hamsters were anesthetized by isoflurane (#R510-22, RWD Life Science) and the nasally inoculated with 1×10^4^ PFU dose of SARS-CoV-2 diluted in 200uL PBS (#10010031, GIBCO). Body weight of these hamsters were measured by electronic balance. Blood was collected for detection of serum neutralizing antibody. Hamsters were treated with isoflurane lightly, after that, capillary tube was used to collect blood from orbital vein. Hamsters were euthanized at indicated time point for detection of viral load and analysis of pathogenesis in lung lobes.

**Dexamethasone Treatment**

Dexamethasone (#HY-14648, MCE) was dissolved in DMSO (#D5879, SIGMA-ALDRICH) at a high concentration (20mg/mL) and stored in ultra-low temperature refrigerator. Hamsters were treated with isoflurane lightly, after that, dexamethasone stock was dissolved in 0.2 mL of sesame oil (#S25527, Shanghai yuanye) for intraperitoneal injection.

**Detection of Viral RNA**

Viral RNA was extracted by using a QIAamp Viral RNA Mini kit (#52906, Qiagen) according to the manufacturer's instructions. The RT-PCR was conducted by using the SLAN-96S Real-Time System (Hongshi, Shanghai, China) with a SARS-CoV-2 RT-PCR Kit from Wantai (Beijing, China). Relative Viral RNA of SARS-CoV-2 ORF1ab gene and NP gene were determined using primers pairs and probes shown in the kit instruction. Viral RNA copies were expressed on a log_10_ scale after normalized to the standard curve obtained by using ten-fold dilutions of a SARS-CoV-2 stock.

**Detection of Cytokine mRNA**

The lung tissues were cleaved into small pieces and soaked in RNAlater (#AM7021, Invitrogen). Total RNAs in lysed lung tissues were extracted with RNeasy Mini kit (#74106, Qiagen) and reverse-transcribed to cDNA with Fast-King Strand cDNA Synthesis Kit (#FP313, TIANGEN, Beijing) Diluted cDNAs (1:10) were quantified using SYBR Green I-based real-time PCR using the LightCycler® 480 instrument (Roche) per manufacturer’s instructions. Threshold cycle (Ct) of each gene was normalized to the internal reference gene (hamster γ-actin) and comparative Ct (2-ΔΔCt) method was utilized to calculate changes in chemokine and cytokine gene expression profile. The gene-specific primers (5’ to 3’) used for RT-PCR were listed in Supplementary Table S3.

**Detection of Antibody Levels in Hamster Serum Samples**

For detection of neutralizing antibody titer, hamster serum samples were gradient diluted and incubated with 100 TCID_50_ of SARS-CoV-2 for one hour. And then, the mix was added into 96-well plate seed with Vero cells for another one-hour incubation. Three days after incubation, the inhibition of cytopathic effects was observed and used for calculation of the serum neutralizing antibody titer. A Hamster SARS-CoV-2 RBD-IgG ELISA Kit (Wantai, Beijing, China) was used to detect the RBD-specific Ig levels in hamster serum samples. In brief, serum samples were diluted and added into 96-well plate coated with SARS-CoV-2 RBD antigen. After incubation, HRP labelled Goat Anti-Syrian Hamster IgG H&L (#ab6892, Abcam) was used as secondary antibody.

**Histopathological Studies**

For pathological analysis, lung tissues were fixed in formalin for more than 48 hours, dehydrated and then embedded in paraffin wax. The wax block of lung tissues was cut into 4μm sections for several pathological staining and analysis. H&E staining was employed for analysis of general lung pathogenic lesions include pulmonary edema, consolidation and inflammation. The standards for pathological score of lung tissues in this study are derived from our previous study in hamster model. Comprehensive pathological score of lung sections were performed according to the degree of lung lesions include alveolar septum hyperplasia, consolidation and impairment of alveolar structure, fluid exudation, mucus suppository, thrombus, inflammation recruitment and infiltration of immune cells in each individual lung lobes. For each hamster, three or four lung lobes were employed for evaluation of comprehensive pathological score. In brief, H&E staining result of each lung lobe was analyzed for its severity of pathological change. The pathological score include: a) Alveolar septum thickening and consolidation; b) Hemorrhage, exudation, pulmonary edema and mucous; c) Recruitment and infiltration of inflammatory immune cells. For each issue, score related to the severity: 0 indicate no pathological change was observed, 1 indicate moderate pathological change, 2 indicate mild pathological change, 3 indicate severe pathological change and 4 indicate very severe pathological change. In conclusion, scores of such three issues were added as the comprehensive pathological score of a lung lobe, and the average comprehensive pathological score of the lobes indicate the severity of lung pathogenesis in an evaluated hamster. The images of whole lung lobes were screened by a high-throughput screening microscope system (EVOS M7000, Invitrogen of Thermo Fisher Scientific).

**Statistical Analysis**

Student’s unpaired two-tailed t-test and one-way ANOVA were performed using GraphPad Prism 8.0 (GraphPad Software). Data are presented as the means ± SD. Two-sided p-values <0.05 were considered significant: *P <0.05, **P <0.01, ***P <0.001, NS indicates no significance.


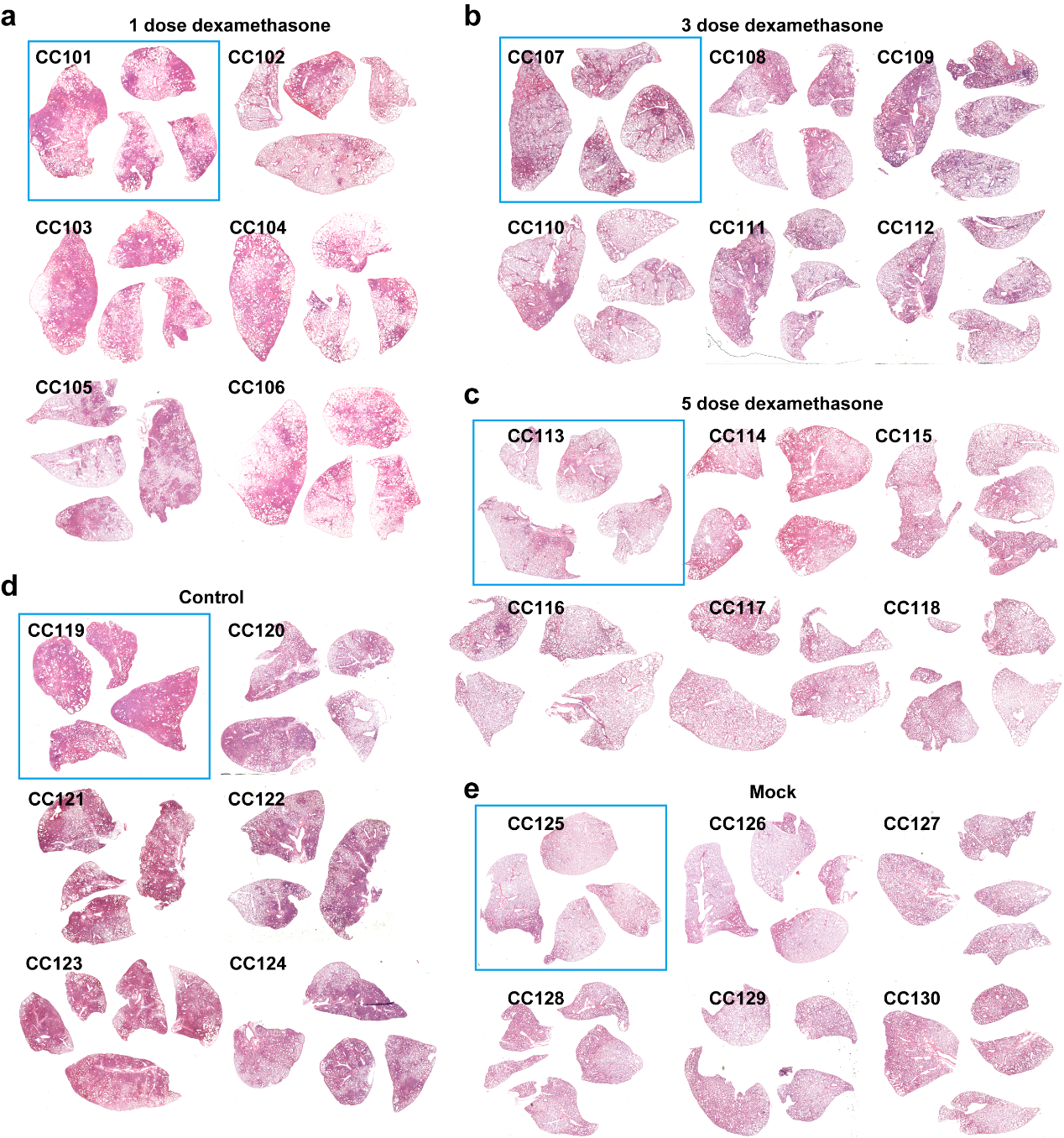


**Supplementary Figure 1. H&E staining of lung lobes collected from hamsters infected with SARS-CoV-2 (AP-8/ancestral strain).** All of the hamsters were sacrificed at 7 dpi. For each hamster, four lung lobes were fixed in formalin for pathological analysis. H&E staining for lung lobe sections collected from SARS-CoV-2 (AP-8/ancestral strain) infected hamsters with **(a)** 1-, **(b)** 3- and **(c)** 5-dose dexamethasone treatment was screened by an auto-microscope system. **(d)** The lung lobe sections collected from SARS-CoV-2 infected hamsters without therapy were set as controls. **(e)** The lung lobe sections collected hamsters without infection were set as mocks. The representative images in blue box were shown in Figure 1c.


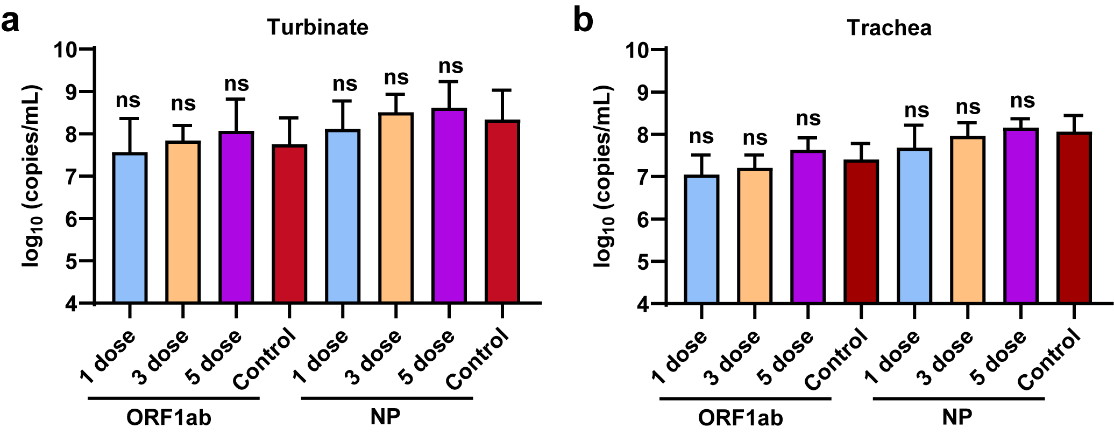


**Supplementary Figure 2. Detection of viral RNA levels in respiratory tract organs.** Viral RNA levels in **(a)** turbinate and **(b)** trachea collected from hamsters at 7 dpi were measured by RT-PCR (n=6). The primers of SARS-CoV-2 ORF1ab and NP genes were used.


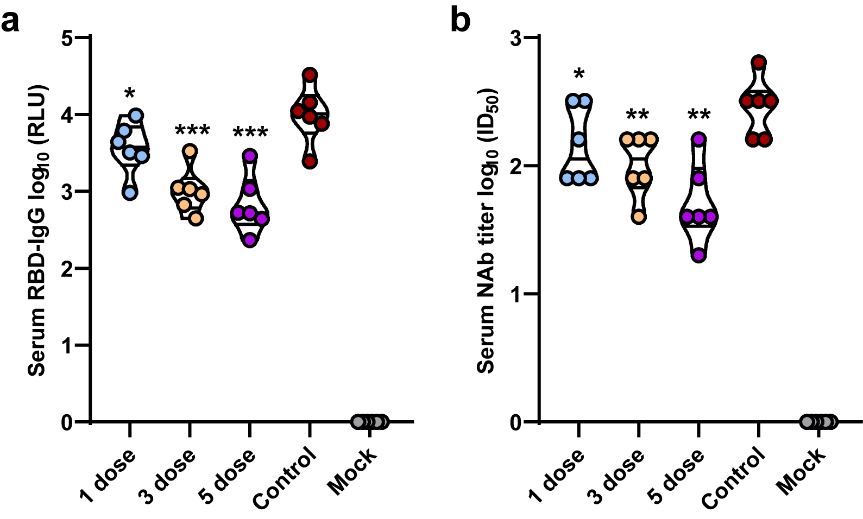


**Supplementary Figure 3. Antibody response in the SARS-CoV-2 infected hamster with dexamethasone treatment.** **(a)** Measurement of SARS-CoV-2 RBD specific antibody levels in hamster serum samples collected at 7 dpi by ELISA (n=6). **(b)** Detection of neutralizing antibody titers of the hamster serum samples collected at 7 dpi by a CPE-based titration method in 96-well plates.


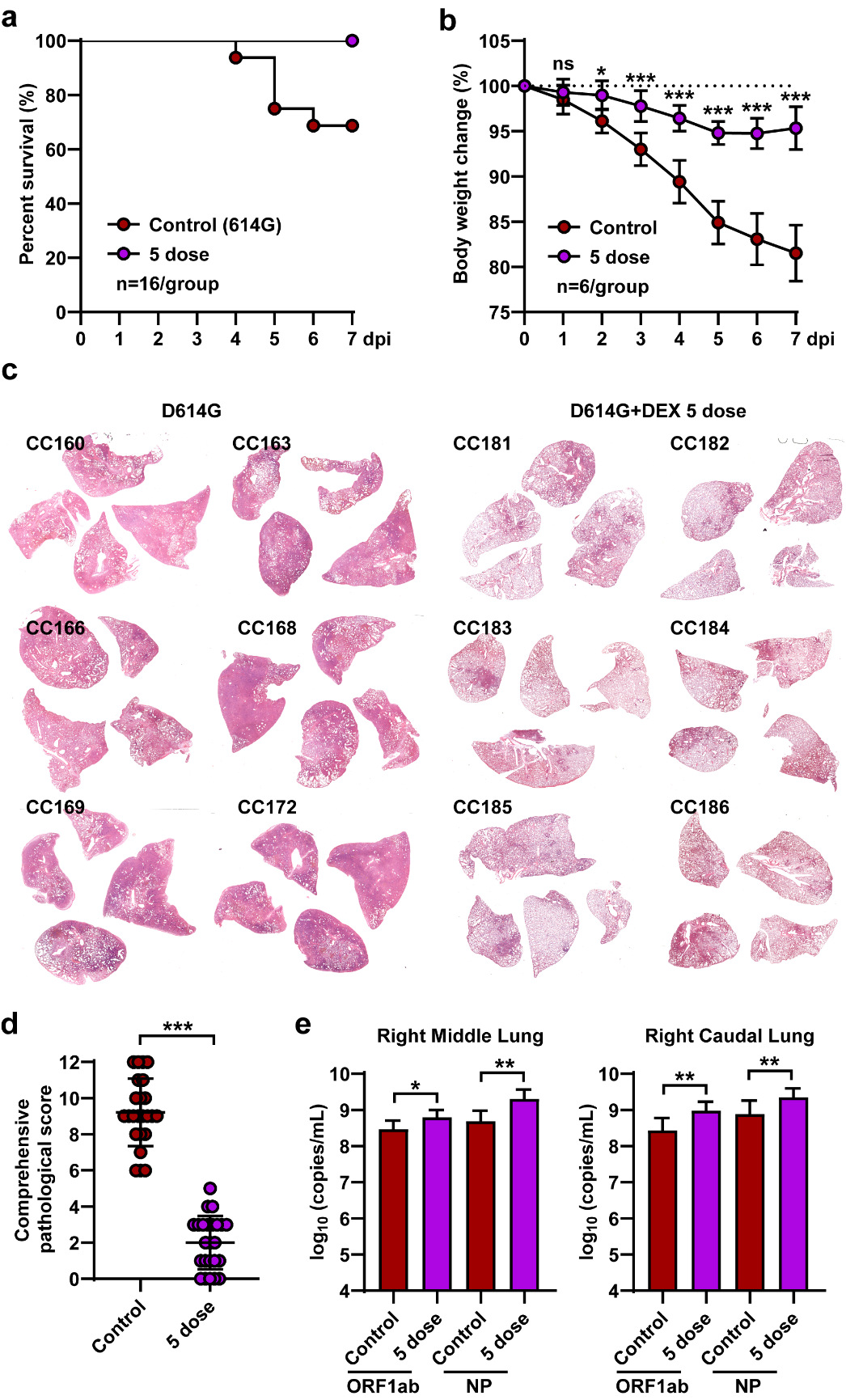


**Supplementary Figure 4. Dexamethasone treatment relieves SARS-CoV-2 variant induced body weight loss, diffuse lung injury and death in hamster.** Male hamsters were intranasally inoculated with 1×10^4^ PFU of SARS-CoV-2 variant 614G virus, and then received intraperitoneal injection of 5 dose of dexamethasone. The 614G virus infected hamsters without treatment were set as control. **(a)** Survival curve (n=16) and **(b)** Body weight changes of 614G virus infected hamster from 0 to 7 dpi (n=6). **(c)** H&E staining for lung lobe sections collected from 614G virus infected hamsters at 7 dpi. **(d)** Comprehensive pathological scores for lung sections were determined based on the severity and percentage of injured areas for each lung lobe. Viral RNA levels in **(e)** right middle lung tissue (near hilum) and right caudal lung tissue (away hilum) collected from hamsters at 7 dpi were measured by RT-PCR (n=6). The primers of SARS-CoV-2 ORF1ab and NP genes were used.


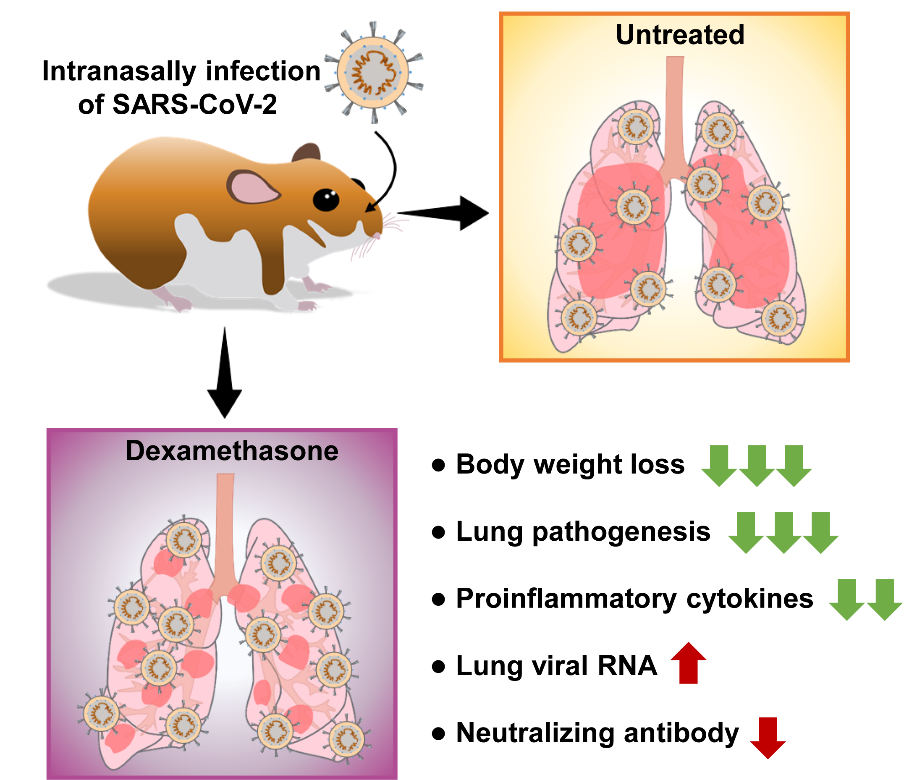


**Supplementary Figure 5. Summary of dexamethasone treatment in hamsters infected with SARS-CoV-2.** This study revealed that dexamethasone is a double-edged sword for therapy of SARS-CoV-2-induced severe pneumonia and death. Dexamethasone is adequate to relieve the diffusion of lung injury by inhibiting the excessive proinflammatory cytokines. However, dexamethasone induces attenuation of antibody response and enhances viral replication in lung tissues.

**Table S1.** Comprehensive pathological score of the hamsters infected with SARS-CoV-2 (AP-8/ancestral strain) sacrificed at 7 dpi.

| **Group** | **Identifier** | **Pathological lesions** | | | **Comprehensive pathological score** |  |
| --- | --- | --- | --- | --- | --- | --- |
|  |  |  |  |  |  |  |
|  |  | **Alveolar septum hyperplasia and consolidation** | **Pulmonary edema, hemorrhage and mucus suppository** | **Recruitment and infiltration of inflammatory cells** |  |  |
|  |  |  |  |  |  |  |
| **1 dose dexamethasone** | **CC101** | 2+1+2+1 | 2+2+1+1 | 3+2+2+2 | 7+5+5+4 |  |
|  | **CC102** | 2+1+1+0 | 2+2+1+1 | 2+1+0+1 | 6+4+2+2 |  |
|  | **CC103** | 3+2+1+1 | 3+2+1+1 | 3+2+1+2 | 9+6+3+4 |  |
|  | **CC104** | 2+0+0+1 | 2+1+1+1 | 3+1+1+2 | 7+2+2+4 |  |
|  | **CC105** | 2+2+0+1 | 2+2+0+1 | 3+2+1+2 | 7+6+1+4 |  |
|  | **CC106** | 2+2+1+1 | 2+1+1+1 | 3+2+1+1 | 7+5+3+3 |  |
| **3 dose dexamethasone** | **CC107** | 2+1+1+1 | 2+2+2+2 | 2+2+2+2 | 6+5+5+5 |  |
|  | **CC108** | 2+2+1+0 | 2+2+2+1 | 2+2+2+0 | 6+5+5+1 |  |
|  | **CC109** | 3+2+1+1 | 3+2+0+0 | 3+3+1+1 | 9+7+2+2 |  |
|  | **CC110** | 2+0+0+0 | 2+1+1+0 | 2+1+1+0 | 6+2+2+0 |  |
|  | **CC111** | 2+1+0+0 | 3+1+0+0 | 3+1+1+1 | 8+3+1+1 |  |
|  | **CC112** | 1+0+0+0 | 2+1+1+1 | 2+1+1+0 | 5+2+2+1 |  |
| **5 dose dexamethasone** | **CC113** | 1+1+0+0 | 1+1+1+0 | 1+1+0+0 | 3+3+1+0 |  |
|  | **CC114** | 1+1+0+0 | 1+1+1+1 | 1+1+0+0 | 3+3+1+1 |  |
|  | **CC115** | 1+1+1+0 | 0+0+0+0 | 0+0+0+0 | 1+1+1+0 |  |
|  | **CC116** | 1+0+0+0 | 1+1+1+0 | 1+1+0+0 | 3+2+1+0 |  |
|  | **CC117** | 1+1+0+0 | 0+0+0+0 | 0+0+0+0 | 1+1+0+0 |  |
|  | **CC118** | 1+1+0 | 0+0+0 | 0+0+0 | 1+1+0 |  |
| **Control** | **CC119** | 4+3+2+2 | 4+3+3+2 | 4+3+3+2 | 12+9+8+6 |  |
|  | **CC120** | 3+2+1+1 | 3+2+2+1 | 3+2+2+1 | 9+6+5+3 |  |
|  | **CC121** | 2+2+2+1 | 3+3+2+1 | 3+3+2+1 | 8+8+6+3 |  |
|  | **CC122** | 2+2+1 | 3+2+2 | 3+2+2 | 8+6+5 |  |
|  | **CC123** | 4+3+3+3+2 | 3+3+3+2+2 | 4+3+3+3+3 | 11+9+9+8+7 |  |
|  | **CC124** | 2+2+1+1 | 3+3+1+1 | 3+2+2+1 | 8+7+4+3 |  |
| **Mock** | **CC125** | 1+0+0+0 | 1+1+0+0 | 0+0+0+0 | 2+1+0+0 |  |
|  | **CC126** | 1+1+0+1 | 0+0+0+0 | 0+0+0+0 | 1+1+0+1 |  |
|  | **CC127** | 0+0+0+0 | 1+1+1+0 | 0+0+0+0 | 1+1+1+0 |  |
|  | **CC128** | 0+0+0+0 | 1+1+0+0 | 0+0+0+0 | 1+1+0+0 |  |
|  | **CC129** | 0+0+0+0 | 1+1+1+0 | 0+0+0+0 | 1+1+1+0 |  |
|  | **CC130** | 0+0+0+0 | 1+1+1+0 | 0+0+0+0 | 1+1+1+0 |  |

**Table S2.** Comprehensive pathological score of the hamsters infected with SARS-CoV-2 (AP-62) sacrificed at 7 dpi.

| **Group** | **Identifier** | **Pathological lesions** | | | **Comprehensive pathological score** |  |
| --- | --- | --- | --- | --- | --- | --- |
|  |  |  |  |  |  |  |
|  |  | **Alveolar septum hyperplasia and consolidation** | **Pulmonary edema, hemorrhage and mucus suppository** | **Recruitment and infiltration of inflammatory cells** |  |  |
|  |  |  |  |  |  |  |
| **614G** | **CC160** | 3+3+3+2 | 4+3+3+3 | 4+3+3+3 | 11+9+9+8 |  |
|  | **CC163** | 3+3+2+2 | 3+3+2+2 | 4+3+2+2 | 10+9+6+6 |  |
|  | **CC166** | 3+3+3+3 | 3+3+3+3 | 3+3+3+3 | 9+9+9+9 |  |
|  | **CC168** | 4+3+3+2 | 4+3+3+2 | 4+3+2+2 | 12+9+8+6 |  |
|  | **CC169** | 4+3+2+2 | 4+3+3+2 | 4+4+3+3 | 12+10+8+7 |  |
|  | **CC172** | 4+4+4+3 | 4+4+3+3 | 4+4+4+4 | 12+12+11+10 |  |
| **614G + 5 dose dexamethasone** | **CC181** | 1+1+0+0 | 1+2+1+0 | 1+1+0+0 | 4+3+1+0 |  |
|  | **CC182** | 1+1+0+0 | 1+1+1+0 | 0+0+0+0 | 2+2+1+0 |  |
|  | **CC183** | 1+1+0+0 | 1+1+0+0 | 1+1+0+0 | 3+3+0+0 |  |
|  | **CC110** | 1+1+1+1 | 1+1+1+1 | 1+1+0+0 | 3+3+1+1 |  |
|  | **CC111** | 1+1+0+0 | 1+1+1+0 | 2+1+0+0 | 4+3+1+0 |  |
|  | **CC112** | 1+1+1+1 | 2+1+1+1 | 2+1+1+0 | 5+3+3+2 |  |

**Table S3.** The gene-specific primers (5’ to 3’) used for RT-PCR for cytokines

| **Genes** | **Forward** | **Reverse** |
| --- | --- | --- |
| **Hamster IFN-γ** | TGTTGCTCTGCCTCACTCAGG | AAGACGAGGTCCCCTCCATTC |
| **Hamster IL-4** | ACAGAAAAAGGGACACCATGCA | GAAGCCCTGCAGATGAGGTCT |
| **Hamster IL-6** | AGACAAAGCCAGAGTCATT | TCGGTATGCTAAGGCACAG |
| **Hamster IL-10** | GGTTGCCAAACCTTATCAGAAATG | TTCACCTGTTCCACAGCCTTG |
| **Hamster IL-13** | AAATGGCGGGTTCTGTGC | AATATCCTCTGGGTCTTGTAGATGG |
| **Hamster TNF-α** | TGAGCCATCGTGCCAATG | AGCCCGTCTGCTGGTATCAC |
| **Hamster γ-actin** | ACAGAGAGAAGATGACGCAGATAATG | GCCTGA ATGGCCACGTACA |
